# The Jerks

**Published:** 1848-09

**Authors:** 


*The Jerks.*—A singular nervous affection has, at times, appeared in some parts of our Western country, analagous to that described by Hecker, in his work on the "Epidemics of the Middle Ages," under the name of "Tarantula," or "Dancing Mania." As the disease is said to be met with occasionally, no apology will be needed for copying from the Ohio Historical Collections, the following graphic description of it:

"In 1803, Austinburgh, Morgan and Harpersfield, experienced a revival of religion, by which about thirty-five from those places united with the church at Austinburgh. This revival was attended with the phenomena of "*bodily exercise*," then common in the West. They have been classified by a clerical writer, as, 1st, the *falling exercises;* 2nd, the *jerking exercises;* 3d, the *rolling exercises;* 4th, the *running exercises;* 5th, the *dancing exercises;* 6th, the *barking exercises;* 7th, the *visions* and *trances*. We make room for an extract from his account of the second of the series, which sufficiently characterizes the remainder.

"It was familiarly called the *jerks,* and the first recorded instance of its occurrence was in East Tennessee, where several hundred, of both sexes, were seized with this strange and involuntary contortion. The subject was instantaneously seized with spasms or convulsions in every muscle, nerve and tendon. His head was thrown or jerked from side to side with such rapidity that it was impossible to distinguish his visage; and the most lively fears were awakened lest he should dislocate his neck, or dash out his brains. His body partook of the same impulse, and was hurried on by like jerks, over every obstacle, fallen trunks of trees, or in a church, over pews and benches, apparently to the most imminent danger of being bruised or mangled. It was useless to attempt to hold or restrain him, and the paroxysm was permitted gradually to exhaust itself. An additional motive for leaving him to himself, was the superstitious notion that all attempts at restraint, was resisting the Spirit of God.

"The first form in which these spasmodic contractions made their appearance, was that of a simple jerking of the arms from the elbows downwards. The jerk was very quick and sudden, and followed with short intervals. This was the simplest and most common form, but the convulsive motion was not confined to the arms—it extended, in many instances, to other parts of the body. When the joint of the neck was affected, the head was thrown backward and forward with a celerity frightful to behold, and which was impossible to be imitated by persons who were not under the same stimulus. The bosom heaved, the countenance was disgustingly distorted, and the spectators were alarmed lest the neck should be broken. When the hair was long, it was shaken with such quickness, backward and forward, as to crack and snap like the lash of a whip. Sometimes the muscles of the back were affected, and the patient was thrown down on the ground, when his contortions for some time resembled those of a live fish, cast from its native element upon the land."

The most graphic description we have, is from one who was not only an eye-witness, but an apologist. He says: "Nothing in nature could better represent this strange and unaccountable operation, than for one to goad another, alternately on every side, with a piece of red hot iron. The exercise commonly began in the head, which would fly backward and forward, and from side to side, with a quick jolt, which the person would naturally labor to suppress, but in vain; and the more any one labored to stay himself and be sober, the more he staggered and the more his twitchings increased. He must necessarily go as he was inclined, whether with a violent dash on lhe ground, and bounce from place to place like a foot-ball, or hop round with limbs or trunk twitching or jolting in every direction, as if they must inevitably fly asunder. And how such could escape without injury, was no small wonder among spectators. By this strange operation, the human form was completely so trensformed and disfigured, as to lose every trace of its natural appearance. Sometimes the head would be twitching right and left, to a half round, with such velocity, and in the quick progressive jerk, it would seem as if the person was transmitted into some other species of creatures. Head-dresses were of but little account among the female jerkers. Even handkerchiefs bound tight round the head would be flirted off almost with the first twitch, and the hair put in the utmost confusion:—this was a very great inconvenience, to redress which, the generality were shorn, though directly contrary to their confession of faith. Such as were seized with the jerks, were wrested at once, not only from under their own government, but that of every one else, so that it was dangerous to attempt confining them or touching them in any manner, to whatever danger they were exposed, yet few were hurt, except it were such as rebelled against the operation, through wilful and deliberate enmity, and refused to comply with the injunctions which it came to enforce.

"From the universal testimony of those who have described these spasms, they appear to have been wholly involuntary. This remark is applicable also to all the other bodily exercises. What demonstrates satisfactorily their involuntary nature is, not only that, as above stated, the twitchings prevail in spite of resistance, and even more for attempts to suppress them; but that wicked men would be seized with them while sedulously guarding against an attack, and cursing every jerk when made. Travellers on their journey, and laborers at their daily work, were also liable to them."—*New York Journal of Medicine.*

